# Bergamot Essential Oil Beverage: Preparation, Formulation Optimization, and Preliminary Evaluation of Antidepressant-like Effects in Mice Induced by Chronic Corticosterone Treatment

**DOI:** 10.3390/foods15101817

**Published:** 2026-05-20

**Authors:** Qingqing Yang, Zhirenyong Zhang, Yan Li

**Affiliations:** 1 College of Food Science and Technology, Huazhong Agricultural University, Wuhan 430070, China; 2023309110105@webmail.hzau.edu.cn (Q.Y.); zhangzry@webmail.hzau.edu.cn (Z.Z.); 2 Key Laboratory of Environment Correlative Dietology, Huazhong Agricultural University, Ministry of Education, Wuhan 430070, China

**Keywords:** bergamot essential oil, beverage, depression, chronic corticosterone model, HPA axis, gut–brain axis

## Abstract

Bergamot essential oil (BEO) has demonstrated antidepressant potential, but its oral application is limited by poor water solubility and undesirable organoleptic properties. In this study, a BEO-loaded beverage was developed based on a whey protein-stabilized oil-in-water emulsion system. The optimal formulation, determined via single-factor experiments combined with orthogonal optimization, consisted of inulin (0.5 g/50 g), milk powder (2.0 g/50 g), sucralose (0.008 g/50 g), and sodium carboxymethyl cellulose (0.04 g/50 g). The resulting beverage remained stable without visible phase separation during 4 months of storage at 4 °C. In a chronic corticosterone treatment (CCT)-induced mouse model of depression, oral administration of the BEO beverage increased activity in the central area of the open field test and exploratory behavior in the elevated plus maze, while reducing repetitive stereotyped behaviors in the marble burying test. At the molecular level, the BEO beverage was associated with reduced levels of interleukin-1β (IL-1β), tumor necrosis factor-alpha (TNF-α), interleukin-6 (IL-6), and corticosteroid (CORT), and increased levels of corticotropin-releasing hormone (CRH), adrenocorticotropic hormone (ACTH), serotonin (5-HT), dopamine (DA), and norepinephrine (NE). Additionally, the BEO beverage was associated with observed alleviation of neuronal damage in the hippocampal CA3 region, upregulation of brain-derived neurotrophic factor (BDNF), improved gut microbial diversity, and altered host metabolic profiles. Collectively, these findings suggest that the BEO emulsion beverage is a feasible intervention for alleviating depression-like behaviors in the mouse model, and provide initial associative evidence supporting its potential as a functional food for mood management.

## 1. Introduction

Depression is a major mood disorder whose global prevalence is steadily rising, making it an urgent public health concern [1]. The disorder is clinically characterized by persistent low mood, anhedonia, social withdrawal, and cognitive impairment, often accompanied by anxiety and suicidal ideation. These symptoms severely affect patients’ quality of life and impose a substantial socioeconomic burden [2]. The pathogenesis of depression is complex and involves multiple pathological processes, including dysregulation of monoamine neurotransmitters, dysfunction of the hypothalamic–pituitary–adrenal (HPA) axis, neuroinflammation, and impaired neuroplasticity [3,4,5]. Given the ever-increasing social stress, the prevention and treatment of depression have become a major public health challenge.

Although commonly used clinical antidepressants, such as selective serotonin reuptake inhibitors (SSRIs), have certain therapeutic effects, they may also cause side effects including weight gain and gastrointestinal discomfort. Some patients may develop drug resistance or dependence [6]. About 40% of patients do not achieve satisfactory outcomes, creating an urgent need for alternative or complementary intervention strategies [3]. In contrast, plant-derived bioactive substances offer multi-target effects and are often considered safer due to their natural origin. However, essential oils are biologically active, and their safety depends on dose, composition, and administration route, warranting careful evaluation. These compounds exert antidepressant effects by regulating neurotransmitters, reducing neuroinflammation, and protecting neurons [7,8,9]. As a result, these natural products are attracting attention for the prevention and treatment of depression. They are now a research hotspot in functional foods.

Essential oils have attracted considerable attention due to their unique physicochemical properties. Their volatile aromatic components can directly stimulate the olfactory system, while their lipophilic small-molecule components can readily cross the blood–brain barrier and thus act rapidly on the central nervous system [10]. Existing studies have primarily focused on the antidepressant and anxiolytic effects of essential oils through aromatherapy [11]. Bergamot essential oil (BEO) is a natural extract obtained from the fruit of Citrus bergamia. It is rich in compounds such as limonene, linalool, and linalyl acetate, and exhibits various biological activities, including anti-inflammatory, antioxidant, and mood-regulating effects [12,13]. Traditionally, BEO has been used in aromatherapy to relieve stress, anxiety, and emotional tension [14,15]. Studies have shown that inhalation of BEO reduces depressive-like behaviors in rodents, attributed to the preservation of hippocampal neuronal plasticity and modulation of the olfactory-limbic neural circuit [16]. Injection of BEO alleviates aluminum-induced anxiety-like behaviors through antioxidant, anti-inflammatory, and GABA-regulating mechanisms [17]. At the cellular level, BEO attenuates corticosterone-induced apoptosis by activating the PI3K/AKT/mTOR signaling pathway and upregulating BDNF and 5-HT expression [18]. In addition to inhalation, oral administration of essential oils allows absorption and metabolic transformation in the gastrointestinal tract, followed by systemic circulation. This produces systemic effects and supports disease prevention and health maintenance [19].

However, existing studies have primarily focused on inhalation or injection routes. The antidepressant potential of orally administered BEO remains largely unexplored. Furthermore, issues such as low water solubility and poor taste limit the application of BEO in functional foods. To address these gaps, this study had two objectives: (1) to develop a whey protein-stabilized emulsion-based BEO beverage that overcomes the limitations of poor water solubility and undesirable taste, with formulation optimized using single-factor tests and an orthogonal design; (2) to provide a preliminary evaluation of this oral BEO formulation in a chronic corticosterone treatment-induced depression mouse model. Exploratory analyses were conducted to investigate associations with neuroinflammation, neurotransmitters, the HPA axis, and the gut microbiota–metabolite axis. This work offers a scientific basis for developing BEO-based functional beverages for mood management.

## 2. Materials and Methods

### 2.1. Materials and Reagents

Bergamot essential oil (BEO, lot No. KS373174, CAS 8007-75-8) was purchased from Shanghai Yuanye Bio-Technology Co., Ltd. (Shanghai, China). A qualified certificate confirmed its three main components—limonene, Linaly acetate, and linalool—made up over 80% of the composition. Whey protein concentrate (WPC, protein content 82.5% in dry) was purchased from Weifeng Biotechnology Co., Ltd. (Zhengzhou, China). Flaxseed oil (FSO) was sourced from Shengmai Co., Ltd. (Benxi, China). Corticosterone (CORT) and fluoxetine hydrochloride (FLU) were obtained from TCI Chemical Co., Ltd. (Tokyo, Japan). Whey powder was purchased from Zhenghong Biotechnology Co., Ltd. (Zhangshu, China). Inulin was procured from Fengning Pingan High-Tech Industry Co., Ltd. (Zhengzhou, China). Sodium carboxymethyl cellulose (CMC) was purchased from Shanghai Changguang Enterprise Development Co., Ltd. (Shanghai, China). Sucralose was obtained from Jiahe Xuri Co., Ltd. (Zhengzhou, China). Unless otherwise stated, all other reagents were of analytical grade.

### 2.2. Animals

Male C57BL/6J mice (6 weeks old, body weight 18–22 g) were purchased and housed at the Laboratory Animal Center of Huazhong Agricultural University (Wuhan, China). All procedures were approved by the Ethics Committee of the Biological Experiment Center of Huazhong Agricultural University (Ethics Approval No. HZAUMO-2026-0054) and followed institutional and national guidelines. Before the experiments, mice were acclimated for 1 week. The facility maintained adequate ventilation, a 12 h light/dark cycle, an ambient temperature of 25 ± 0.5 °C, and a relative humidity of 50 ± 5%.

### 2.3. Preparation of BEO Beverage

WPC was dispersed in ultrapure water under magnetic stirring and left to hydrate overnight at 4 °C to obtain a fully hydrated 5 wt% WPC solution. The oil phase was prepared by mixing BEO with FSO. Based on preliminary experimental results, the BEO content in the oil phase was set at 50 wt%. To combine the two phases, the aqueous WPC solution and the oil phases were mixed together at a volume ratio of 9:1. This mixture was then pre-emulsified using a high-speed shear homogenizer (PD500, Prima Technology Group Co., Ltd., London, UK) at 10,000 rpm for 5 min to produce a coarse emulsion. The emulsion was subsequently subjected to ultrasonication at 300 W for 15 min at 4 °C to further reduce droplet size and enhance emulsion stability. Then, 10 g of the emulsion was weighed and diluted 5 times with water. Milk powder, inulin, sodium carboxymethyl cellulose, and sucralose were added sequentially and mixed thoroughly. The mixture was homogenized four times using a high-pressure microfluidizer (M-110L, Microfluidics Co., Ltd., Westwood, MA, USA) at 12,000 psi, followed by sterilization at 121 °C. The resulting BEO beverage was stored at 4 °C for subsequent use. For convenience, this beverage is referred to as BEO in the following text.

### 2.4. Single-Factor Experimental Design

To investigate the effects of different ingredient additions on beverage quality, a single-factor experimental design was used. The addition levels of inulin, milk powder, CMC, and sucralose were selected as the investigated factors. Under the condition of fixing other basic formulation components, the addition levels for each factor were set as follows: inulin (g/50 g): 0.5, 1.0, 1.5, 2.0, 2.5; milk powder (g/50 g): 1.0, 2.0, 3.0, 4.0, 5.0; CMC (g/50 g): 0.02, 0.04, 0.06, 0.08, 0.10; sucralose (g/50 g): 0.004, 0.008, 0.012, 0.016, 0.020. By varying the addition level of a single factor while keeping the levels of the other factors constant, the influence of each factor on sensory evaluation (Table 1) was assessed to determine the optimal addition range for each factor.

Sensory evaluation was conducted in a sensory analysis room, and each experiment was repeated three times. The sensory panel consisted of 10 trained evaluators (5 males, 5 females, age 22–30 years) from the Key Laboratory of Environment Correlative Dietology, Huazhong Agricultural University. All evaluators had no history of taste or smell disorders and were asked to refrain from eating or drinking for 1 h before testing. The evaluation criteria were shown in Table 1, with four attributes assessed: appearance (20 points), mouthfeel (30 points), odor (20 points), and taste (30 points), giving a total possible score of 100 points. For each sample, evaluators were instructed to observe the appearance first, then smell the odor, and finally take a small amount (approximately 10 mL) into the mouth, hold it for 10 s to assess mouthfeel and taste, and then expectorate. Between samples, evaluators rinsed their mouths thoroughly with purified water and waited 60 s before proceeding to the next sample. Samples were coded with random three-digit numbers and presented in random order to each evaluator. All samples were evaluated at room temperature (25 °C). The final score for each sample was calculated as the mean ± standard error of the total scores from all evaluators.

### 2.5. Orthogonal Array Design

Based on the single-factor experiments, an orthogonal array design with four factors and three levels was adopted for optimization. Sensory score was used as the evaluation index to determine the optimal formulation of the BEO beverage. The factors and levels of the orthogonal array design are shown in Table 2. The optimal formulation was selected directly from the orthogonal test results without a separate confirmatory experiment.

### 2.6. Storage Stability Evaluation

The freshly prepared BEO beverage was dispensed into glass bottles and stored at 4 °C in the dark. The particle size and zeta potential of the protein emulsion were measured on days 1, 7, 14, 30, 60, and 120 to evaluate the storage stability of the protein emulsion.

The mean particle size and particle size distribution of beverages were determined using a laser particle size analyzer (Mastersizer 2000, Malvern, Worcestershire, UK). During the measurement, the refractive indices of the dispersed phase and continuous phase were set to 1.474 and 1.330, respectively.

The zeta potential of beverages was measured using a nanoparticle size and zeta potential analyzer (Malvern Instruments, Worcestershire, UK). All measurements were conducted at 25 °C. Prior to analysis, the beverages were diluted with ultrapure water to an oil phase concentration of 0.01 wt% to avoid multiple light scattering effects.

### 2.7. Animal Experiment

#### 2.7.1. Model Establishment and Animal Grouping

Forty mice were randomly assigned into five groups (*n* = 8 each): control group (CON), model group (chronic corticosterone treatment, CCT), vehicle group (VEH), positive control group (FLU), and BEO beverage group (BEO). The sample size of *n* = 8 per group was based on previous similar studies [20], which have shown that this sample size is sufficient to detect statistically significant differences in behavioral tests. Specifically, mice were individually selected from the pool and allocated to groups sequentially without bias. Predefined exclusion criteria were: (1) death; (2) severe illness (e.g., weight loss > 25% of initial body weight, hunched posture, or lethargy); and (3) gavage errors. None of these criteria were met, and all 40 mice were included in the final analysis. All mice underwent a 9-day acclimation by oral gavage with 200 μL drinking water daily. Subsequently, a 21-day formal experiment was conducted. All groups except CON received CORT (0.1 mg/mL in 1% ethanol water) as the only drinking water to induce a depression; The CON group had water with 1% ethanol. Daily, the CON and CCT groups received 200 μL drinking water by gavage; the VEH group received 200 μL beverage matrix without BEO; the FLU group received fluoxetine (20 mg/kg); and the BEO group received 200 μL BEO beverage, providing a daily dose of approximately 2 mg BEO per mouse, equivalent to approximately 100 mg/kg body weight. Body weight was recorded every three days.

#### 2.7.2. Behavioral Tests

Mice were randomly selected for the tests, and the evaluators were blinded to the group assignments during behavioral assessments. The tests were performed in the following order: open field test (OFT) first, followed by elevated plus maze (EPM) 24 h later, and marble burying test (MBT) another 24 h later, allowing animals to recover from handling between tests.

OFT: Each mouse was placed individually in the center of an open field equipment (50 × 50 × 40 cm) and allowed to explore freely for 5 min. An automated video-tracking system recorded total movement distance, the proportion of movement in the central area, and immobility time. After each trial, the equipment was thoroughly cleaned with 70% (*V*/*V*) ethanol to eliminate olfactory cues.

EPM: The maze consisted of two open arms (35 × 5 cm), two closed arms (35 × 5 × 10 cm), and a central platform (5 × 5 cm). Mice were placed on the central platform facing an open arm, and their activity was recorded for 5 min. The frequency of open-arm entries and cumulative time spent in the open arms were quantified. The maze was cleaned with 70% (*V*/*V*) ethanol between tests.

MBT: Twenty glass marbles (16 mm diameter) were evenly arranged for a 4 × 5 array on the bedding of a testing cage. Each mouse was placed in a corner of the cage, and after 30 min the number of buried marbles was recorded. A marble was considered buried when at least 2/3 of its surface was covered by bedding material.

#### 2.7.3. Neurotransmitter Analysis

Samples were extracted with 20% acetonitrile/methanol, and the supernatants were subjected to analysis using an LC–MS/MS system (ExionLC AD–QTRAP 6500+, SCIEX, Framingham, MA, USA). Chromatographic separation was performed on a Waters ACQUITY UPLC HSS PFP column (100 mm × 2.1 mm, 1.8 μm) with a mobile phase consisting of 0.1% formic acid in water (A) and acetonitrile (B) under gradient elution at 0.35 mL/min. The column temperature was maintained at 40 °C, and the injection volume was 2 μL. Mass spectrometric detection employed an ESI Turbo Ion-Spray source operated in both positive and negative modes using multiple reaction monitoring (MRM). Metabolite identification was achieved by comparison with the Metware Database based on authentic standards, followed by peak integration and quantification using standard curves.

#### 2.7.4. Enzyme-Linked Immunosorbent Assay (ELISA)

Serum levels of inflammatory cytokines (TNF-α, DB049-Mu; IL-1β, DB1074-Mu; IL-6, DB1061-Mu) were measured using ELISA kits (Huding Biotechnology Co., Ltd., Shanghai, China), while serum levels of CORT (E-OSEL-M0001) and adrenocorticotropic hormone (ACTH, E-EL-M0079) were measured using ELISA kits (Elabscience Biotechnology Co., Ltd., Wuhan, China). Hypothalamic tissues were homogenized in PBS containing 1 mM PMSF at a ratio of 1:9 (*w*/*v*). After centrifugation at 5000× *g* for 10 min at 4 °C, corticotropin-releasing hormone (CRH) levels in hypothalamic supernatants were quantified by ELISA (E-EL-M0351, Elabscience Biotechnology Co., Ltd., China). Optical density was read at 450 nm using a Multiskan SkyHigh microplate spectrophotometer (Thermo Scientific, Waltham, MA, USA), and concentrations were calculated from standard curves.

#### 2.7.5. Histological and Immunofluorescence Analysis

The isolated tissues were fixed in 4% paraformaldehyde for 24 h, dehydrated through graded ethanol, cleared in xylene, and embedded in paraffin. Sections approximately 4 μm thick were prepared. For hematoxylin–eosin (HE) staining and Nissl staining, sections were deparaffinized, rehydrated, stained with hematoxylin–eosin or toluidine blue, and mounted with neutral resin. For immunofluorescence staining, antigen retrieval was performed in 0.01 M citrate buffer (pH 6.0). Endogenous peroxidase activity was blocked with 3% H_2_O_2_ for 10 min at room temperature, followed by blocking with rabbit serum. Sections were then incubated overnight at 4 °C with primary antibodies against BDNF (1:800, 28205-1-AP, Proteintech, Rosemont, IL, USA) and NeuN (1:500, BA1073, Biossci, Wuhan, China). After equilibration to room temperature, HRP-conjugated secondary antibodies (Alexa Fluor 488 and 647, 1:400, Invitrogen, Eugene, OR, USA) were applied for 1 h in the dark. Nuclei were counterstained with DAPI (1:500), and sections were mounted with antifade medium. Whole-slide images were acquired using a Pannoramic Scan II digital pathology scanner (3DHISTECH, Budapest, Hungary).

#### 2.7.6. 16S rRNA Gene Sequencing

Following euthanasia, intestinal contents were collected into cryogenic tubes and snap-frozen in liquid nitrogen. Genomic DNA was extracted using the SDS method, and its purity and concentration were verified by agarose gel electrophoresis. DNA was diluted to 1 ng/μL. The V3–V4 hypervariable region of the bacterial 16S rRNA gene was amplified using the primer pair 341F-806R with Phusion High-Fidelity PCR Master Mix (Thermo Fisher Scientific, Waltham, MA, USA). After verification and purification of PCR products, equimolar amounts were pooled, and target bands were recovered. Libraries were constructed using the TruSeq DNA PCR-Free Sample Preparation Kit (Illumina, CA, USA) and quantified by Qubit and qPCR before sequencing on a NovaSeq 6000 system (Illumina, CA, USA). Bioinformatic analysis was performed using Metware Cloud (https://cloud.metware.cn).

#### 2.7.7. Untargeted Metabolomics Analysis

Samples were thawed on ice and extracted with 70% methanol/water containing internal standards. After vortexing, sonication, low-temperature precipitation, and centrifugation, supernatants were subjected to LC-MS analysis using a Vanquish–Q Exactive HF-X system (Thermo Scientific, Waltham, MA, USA). Separation was achieved on a Waters ACQUITY Premier HSS T3 column (2.1 × 100 mm, 1.8 μm) with a mobile phase of 0.1% formic acid in water (A) and acetonitrile (B) under gradient elution at 0.4 mL/min. The column temperature was maintained at 40 °C, and the injection volume was 4 μL. Mass spectrometry was conducted in both positive and negative ion modes with full-scan acquisition over an m/z range of 75–1000 at a resolution of 35,000. Data processing and analysis were performed using Metware Cloud.

### 2.8. Statistical Analysis

All tests were independently repeated at least three times. Data were analyzed using IBM SPSS Statistics 26 software. Results were expressed as mean ± standard error of the mean (SEM). Prior to conducting one-way analysis of variance (ANOVA), the normality of the data was assessed using the Shapiro–Wilk test. The differences between the samples were evaluated using ANOVA and Duncan’s multiple comparisons. A threshold of *p* < 0.05 was established for statistical significance. Visualization of experimental data was performed using Origin 2024 software.

## 3. Results

### 3.1. Single-Factor Experiments

**Figure 1:**
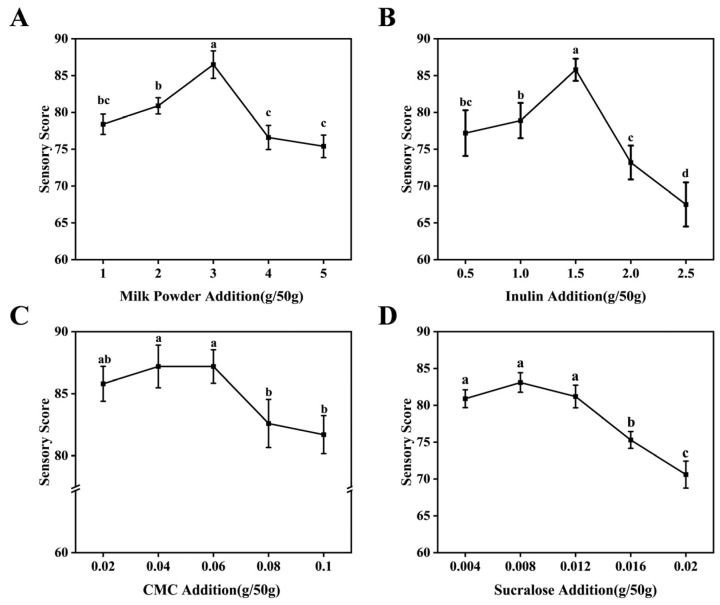
Single-factor sensory evaluation scores. (**A**) Effect of inulin addition on sensory score. (**B**) Effect of milk powder addition on sensory score. (**C**) Effect of CMC addition on sensory score. (**D**) Effect of sucralose addition on sensory score. Diverse alphabetical forms denote significant differences, *p* < 0.05.

To investigate the effects of different ingredient additions on sensory scores, various addition levels of inulin, milk powder, CMC, and sucralose were tested individually while keeping the other factors constant. As shown in Figure 1, the sensory score for each ingredient increased initially because the added amount improved a specific characteristic, but then decreased as excessive amount led to negative effects. Specifically, Inulin achieved the highest score at 1.5 g/50 g, this is because an appropriate amount improved mouthfeel and texture, while excessive inulin caused excessive viscosity. Milk powder scored best at 3.0 g/50 g, as a suitable amount enhanced the milky flavor and richness, but excess introduced a milky off-flavor. CMC was optimal at 0.06 g/50 g, an appropriate level improved stability and a smooth texture, but too much resulted in a heavy mouthfeel. Sucralose reached the highest score at 0.012 g/50 g, since a suitable amount balanced the flavor, while excess led to an imbalance in sweetness.

### 3.2. Orthogonal Experiment

To optimize the formulation of the BEO beverage, a four-factor, three-level orthogonal array was used based on results from single-factor experiments. Sensory score was used as the evaluation index. As shown in Table 3, when the sensory score was used as the evaluation index, Run No. 2 exhibited the highest score, with the corresponding optimal factor level combination being A1 (inulin addition) B2 (CMC addition) C2 (sucralose addition) D2 (milk power addition). The order of influence of the four factors on the sensory score was determined as C (sucralose addition) > B (CMC addition) > D (milk powder addition) > A (inulin addition). Consequently, the optimal formulation conditions were determined as follows: inulin at 0.5 g/50 g, milk powder at 2.0 g/50 g, sucralose at 0.008 g/50 g, and CMC at 0.04 g/50 g.

### 3.3. Storage Stability

**Figure 2:**
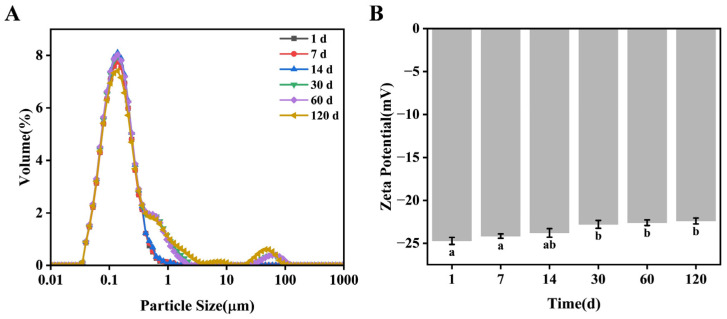
Stability of BEO beverage during storage. (**A**) Particle size distribution. (**B**) Zeta potential. Different letters represent the significant differences (*p* < 0.05) in the performance of the BEO beverage at different storage times.

To evaluate the physical stability of the BEO beverage prepared with the optimal formulation, its particle size distribution and zeta potential were measured at different time points (Figure 2). During the initial storage period (days 0–14), the particle size distribution of the BEO beverage exhibited a typical unimodal distribution, indicating uniform particle size and good dispersibility, with no significant aggregation or flocculation. This is a characteristic feature of good physical stability in emulsion systems [21,22]. As storage time extended to day 30, the particle size distribution began to change significantly, transforming from unimodal to multimodal, with new peaks appearing in the larger particle size range. This observation suggests that partial particle aggregation or coalescence occurred during long-term storage, leading to increased particle size and an uneven distribution, as well as reduced system uniformity [23]. With prolonged storage time, the absolute value of the zeta potential of the BEO beverage showed a gradual decrease. A decrease in the absolute zeta potential value indicates weakened electrostatic repulsion between particles, making them more prone to aggregation and thereby affecting the long-term stability of the emulsion [24]. But the appearance of BEO beverages had no change, indicating that their stability was still acceptable after 4-month storage.

### 3.4. Effect of BEO Beverage on Body Weight of Mice

**Figure 3:**
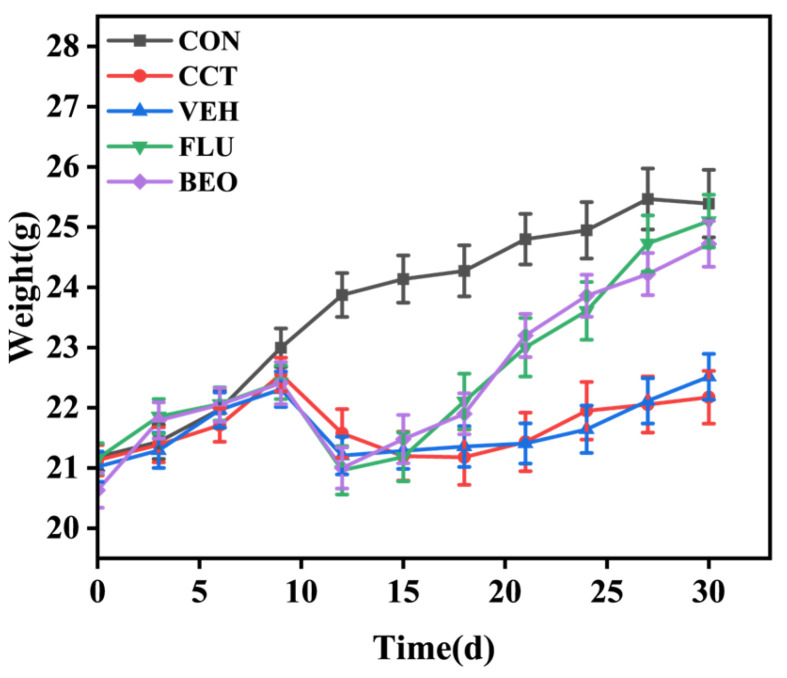
The effect of bergamot essential oil beverage on the body weight of mice. Values represent as mean ± SEM, *n* = 8. CON, control group; CCT, chronic corticosterone treatment group; VEH, vehicle group; FLU, fluoxetine hydrochloride; BEO, Bergamot essential oil group.

To evaluate the effects of BEO beverage on body weight in mice, body weights were recorded every three days for each group. Figure 3 shows that before modeling (days 0–9), initial body weights did not differ among groups. After 21 days of CCT intervention, body weight changes differed significantly. The CCT group had significantly lower body weight than the CON group (*p* < 0.05). The VEH group, which received only the vehicle solution without the active BEO ingredient, also exhibited reduced body weight similar to the CCT group, indicating the vehicle itself had no significant impact on weight. Chronic CCT exposure often reduces appetite and body weight loss in rodents, key indicators of depression-like models [25]. Post-modeling, the BEO beverage group’s body weight was significantly higher than the CCT group (*p* < 0.05) and nearly matched that of the CON group, indicating that the BEO beverage intervention alleviated CCT-induced weight loss and may benefit depression-related physiological dysregulation.

### 3.5. Effect of BEO Beverage on Behavioral Performance in Mice

The OFT, EPM, and MBT were used to evaluate locomotor activity, anxiety-like behavior, and repetitive behavior, respectively, collectively reflecting a depression-like behavioral profile in mice. These methods all rely on the innate conflict in rodents between the desire to explore and the aversion to open or brightly lit environments [26,27,28].

- OFT

**Figure 4:**
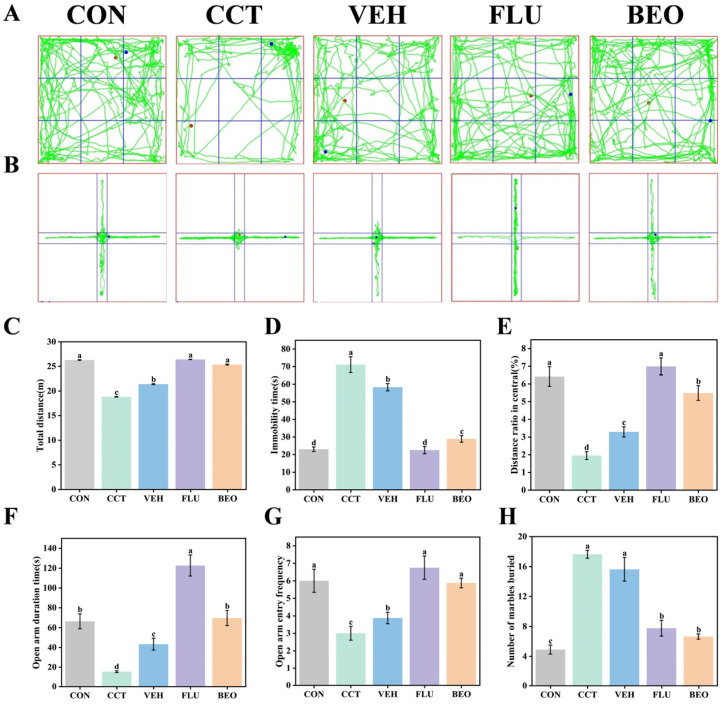
Behavioral experiments in mice. (**A**) Movement locus in OFT. (**B**) Movement locus in EPM. (**C**) Total distance, (**D**) immobility time, and (**E**) distance ratio in central in OFT. (**F**) Open arm duration time, and (**G**) open arm entry frequency in EPM. (**H**) Number of marbles buried in MBT. Values represent as mean ± SEM, *n* = 8. Diverse alphabetical forms denote significant differences, *p* < 0.05. CON, control group; CCT, chronic corticosterone treatment group; VEH, vehicle group; FLU, fluoxetine hydrochloride; BEO, Bergamot essential oil group.

The OFT results (Figure 4A,C–E) showed that compared with the CON group, the CCT and VEH groups exhibited a significant decrease in total distance traveled (*p* < 0.05), a significant increase in immobility time (*p* < 0.05), and a significant reduction in the Distance ratio in central (*p* < 0.05). Their trajectory maps indicated that their activity was mainly concentrated in the peripheral zone, suggesting that chronic CCT exposure induced marked suppression of spontaneous activity and anxiety-like behavior [29]. In contrast, the FLU and BEO intervention groups showed significant reversal of the above parameters (*p* < 0.05), with their activity range expanding toward the central area.

- EPM

The EPM results (Figure 4B,E–G) revealed that the time spent and the number of entries into the open arms were significantly lower in the CCT and VEH groups than in the CON group (*p* < 0.05). Their trajectory maps showed that they primarily stayed in the closed arms, indicating pronounced fear-avoidant behavior toward open environments in the model mice [30]. These parameters were significantly higher in the BEO group than in the CCT group (*p* < 0.05), indicating a marked alleviation of open arm avoidance behavior.

- MBT

The MBT results (Figure 4H) showed that the number of marbles buried by the CCT group was significantly higher than that of the CON group (*p* < 0.05), suggesting increased repetitive and stereotyped behavior, which is a typical manifestation of depressive-like phenotype [31]. The number of marbles buried in the BEO group was significantly lower than that in the CCT group (*p* < 0.05).

Collectively, these results indicate that the BEO beverage was associated with improvements in depression-like behaviors.

### 3.6. Effects of BEO Beverage on Systemic Inflammation, Neurotransmitters, HPA Axis Function, Neuroplasticity and Hippocampal Tissue in Mice

To evaluate the physiological effects of BEO beverage on improving depression-like behaviors, levels of inflammatory cytokines, monoamine neurotransmitters, HPA axis-related hormones, and hippocampal neuroplasticity were measured.

**Figure 5:**
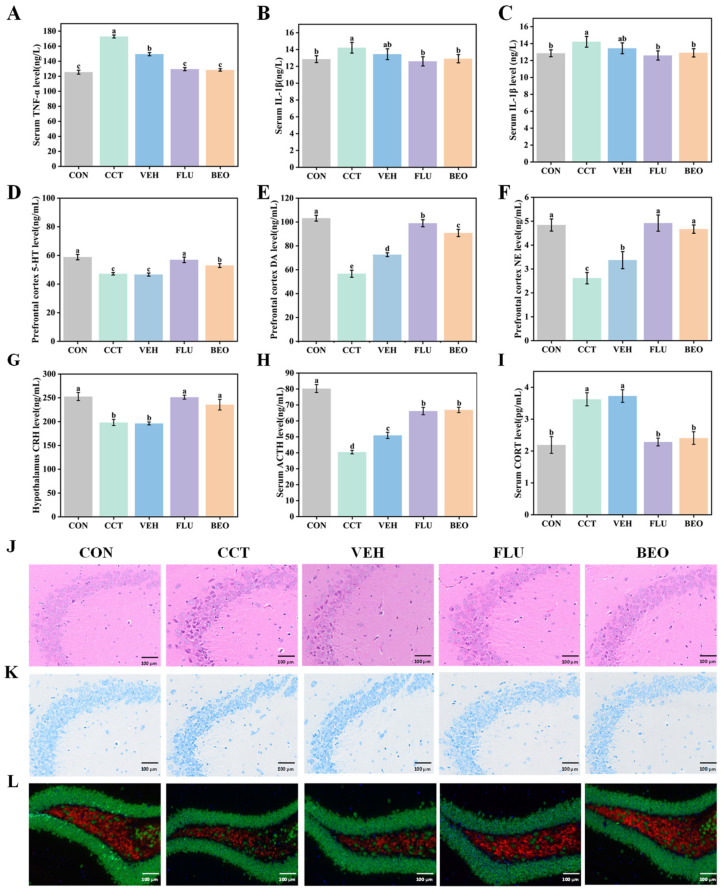
(**A**) Serum TNF-α levels. (**B**) Serum IL-β levels. (**C**) Serum IL-6 levels. (**D**) Prefrontal cortex 5-HT levels. (**E**) Prefrontal cortex DA levels. (**F**) Prefrontal cortex NE levels. (**G**) Hypothalamus CRH levels. (**H**) Serum ACTH levels. (**I**) Serum CORT levels. (**J**) HE staining of brain tissue sections. (**K**) Nissl staining of brain tissue sections. (**L**) Immunofluorescence staining of brain tissue sections, where blue (DAPI) indicates nuclei, green (FITC) represents NeuN, and red (CY5) marks BDNF. Data are represented as mean ± SEM, *n* = 8. Different lowercase letters indicate significant differences, *p* < 0.05. CON, control group; CCT, chronic corticosterone treatment group; VEH, vehicle group; FLU, fluoxetine hydrochloride; BEO, Bergamot essential oil group.

Inflammatory cytokines (Figure 5A–C). Serum levels of IL-1β, TNF-α, and IL-6 in the CCT group were significantly elevated (*p* < 0.05), indicating that chronic CCT exposure induced systemic inflammation. Following the BEO beverage intervention, the levels of these cytokines were significantly reduced (*p* < 0.05) and returned to near-normal levels, demonstrating the anti-inflammatory effect of BEO.

Neurotransmitters (Figure 5D–F). Measurements revealed that the levels of 5-HT, DA, and NE in the prefrontal cortex were significantly decreased in the CCT group (*p* < 0.05). Intervention with FLU or the BEO beverage significantly increased these neurotransmitter levels compared with the CCT group (*p* < 0.05), indicating a negative correlation between monoamine levels and the severity of depressive behavior [32].

HPA axis-related hormone (Figure 5G–I). Compared with the CON group, the CCT group exhibited significantly reduced levels of hypothalamic corticotropin-releasing hormone (CRH) and serum adrenocorticotropic hormone (ACTH), while serum corticosterone (CORT) levels were significantly increased (*p* < 0.05). These results indicate that CCT leads to endogenous HPA axis dysfunction via a negative feedback inhibition mechanism [33]. Following the BEO beverage intervention, the levels of these hormones returned to values close to those of the normal control group, suggesting that BEO beverage was associated with normalization of HPA axis-related parameters.

Hippocampal neuroplasticity (Figure 5J–L). Histopathological observations revealed that HE staining showed reduced neuron number, disordered arrangement, and cell atrophy in the hippocampal CA3 region of the CCT group. Nissl staining confirmed a significant reduction in neuron density and in the number of Nissl bodies. Immunofluorescence staining showed decreased BDNF expression [34]. After the BEO beverage intervention, hippocampal neuronal damage was alleviated, neuronal morphology became more regular, and BDNF expression was upregulated.

In conclusion, the BEO beverage was associated with changes in inflammatory responses, monoamine neurotransmitter levels, HPA axis function, and hippocampal neuronal plasticity, which may contribute to the amelioration of chronic CCT-induced depression-like behaviors in mice.

### 3.7. Effect of BEO Beverage on Gut Microbiota in Mice

**Figure 6:**
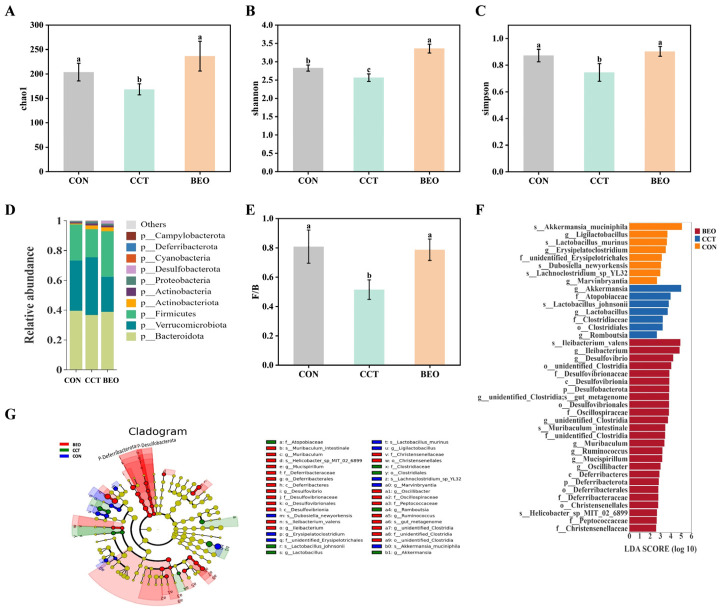
The influence of BEO beverages on the intestinal flora of mice. (**A**) Chao1 index. (**B**) Shannon index. (**C**) Simpson index. (**D**) The differences in the intestinal microbial communities at the phylum level among the various groups. (**E**) Ratio of relative abundance of *Firmicutes* and *Bacteroidota*. (**F**) LEfSe analysis among groups. (**G**) Differential microbial tree diagram. Each small circle represents a category under that level, and its diameter is proportional to the relative abundance. Species with no significant difference are colored yellow; species with differences are colored according to groups, with red, green, and blue nodes representing the groups in which the groups play an important role. If a group is missing, it indicates that there are no significant difference species in that group. The names of the species represented by the letters in the figure are shown in the legend on the right. Different lowercase letters represent significant differences. Values represent as mean ± SEM, *p* < 0.05, *n* = 6. CON, control group; CCT, chronic corticosterone treatment group; BEO, Bergamot essential oil group.

To evaluate the regulatory effect of the BEO beverage on gut microbiota, the composition of the intestinal microbial community was compared among the CON, CCT, and BEO groups. Alpha diversity indices were used to assess microbial richness and diversity. Specifically, the Chao1 index reflected species richness, while the Shannon and Simpson indices characterized community diversity [35]. As shown in Figure 6A–C, the Chao1, Shannon, and Simpson indices were significantly decreased in the CCT group compared with the CON group (*p* < 0.05), indicating that chronic CCT exposure induced a reduction in gut microbiota diversity, which could be associated with impaired gut health. Following BEO intervention, these alpha diversity indices increased, suggesting that BEO partially restored the CCT-induced reduction in microbial richness, which may support the recovery of gut microbiota balance. At the phylum level, the abundance ratio of Firmicutes to Bacteroidota (F/B ratio) was further analyzed (Figure 6D). The F/B ratio has been used as one of several ecological metrics to describe gut microbiota composition, although its reliability as a standalone biomarker of gut health has been debated [36,37,38]. As shown in Figure 6E, CCT decreased the F/B ratio, whereas BEO beverage intervention significantly increased the F/B ratio to a level close to that of the CON group, suggesting a shift toward the control group profile.

Linear discriminant analysis effect size (LEfSe) analysis was performed to identify bacterial taxa specifically enriched in each group (LDA > 2.5, *p* < 0.05). A total of 40 bacterial clades with significant differences were identified across the groups (Figure 6F,G). Specifically, the CON group harbored 8 enriched taxa, including *Akkermansia muciniphila*, *Ligilactobacillus* and *Lactobacillus murinus*, among others. The CCT group exhibited 7 enriched taxa, including *Akkermansia*, *Atopobiaceae*, *Lactobacillus johnsonii*, and *Clostridiaceae*, among others. The BEO group showed 25 enriched taxa, including *Ileibacterium valens*, *Ileibacterium*, *Desulfovibrio*, and *Desulfovibrionaceae*, among others. These results indicate that CCT-treatment was associated with significant alterations in gut microbiota structure, accompanied by a reduced number of enriched taxa. Following BEO beverage intervention, this pattern was associated with an increased number of enriched taxa. However, these findings demonstrate associations rather than causal relationships. Further studies are needed to determine the functional significance of these microbial changes and their potential relationship with depressive-like behavior.

### 3.8. Effect of BEO Beverage on Metabolites in Mice

**Figure 7:**
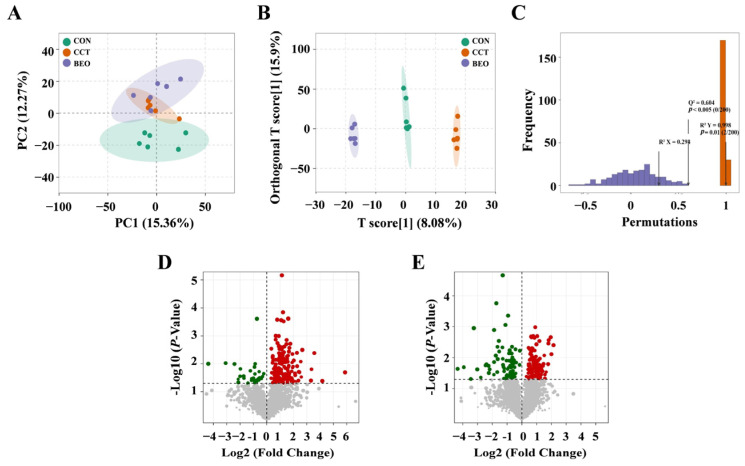
The influence of BEO beverages on the metabolites of mice. (**A**) Principal Component Analysis (PCA) of metabolites. (**B**) OPLS-DA score plot. (**C**) OPLS-DA permutation test. Orange represents the random grouping model R^2^Y, purple represents the random grouping model Q^2^, and the black arrow indicates the values of the original model’s R^2^X, R^2^Y, and Q^2^. (**D**) Volcano plot of CCT group vs. CON group. (**E**) Volcano plot of CCT group vs. BEO group. Red points indicated differentially upregulated metabolites, green points indicated differentially downregulated metabolites, and gray points represented that metabolites did not meet the criteria for differential significance. N = 6. CON, control group; CCT, chronic corticosterone treatment group; BEO, Bergamot essential oil group.

To evaluate the effect of the BEO beverage on the metabolic profile of CCT model mice, non-targeted metabolomics analysis was performed to assess serum metabolic profiles in each group. The principal component analysis (PCA) score plot showed significant differences in metabolic profiles among the CON, CCT, and BEO groups (Figure 7A). Orthogonal partial least squares discriminant analysis (OPLS-DA) further confirmed these inter-group differences (Figure 7B). Permutation test results (Figure 7C) indicated good model stability (R^2^Y = 0.998, Q^2^ = 0.605 > 0.5). Differential metabolites were screened using the criteria of variable importance in projection (VIP) > 1.0 and *p* < 0.05 (Figure 7D,E). Compared with the CON group, a total of 229 differential metabolites were identified in the CCT group, of which 196 were upregulated, and 33 were downregulated. Compared with the CCT group, the BEO intervention group identified 216 differential metabolites, including 134 upregulated and 82 downregulated.

**Figure 8:**
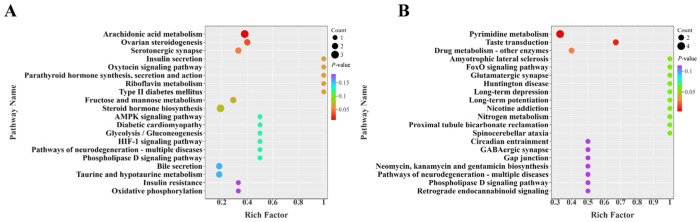
KEGG pathway enrichment analysis. (**A**) CON group vs. CCT group; (**B**) BEO group vs. CCT group.

To further elucidate the biological processes involved in the differential metabolites, KEGG pathway enrichment analysis was performed on the differentially abundant metabolites between groups (Figure 8). Comparison between the CON and CCT groups revealed that the differentially abundant metabolites were significantly enriched in several metabolic pathways, including arachidonic acid metabolism, ovarian steroidogenesis, and the serotonergic synapse. Among these, the arachidonic acid metabolism pathway is a key regulatory pathway of inflammatory responses and is closely associated with neuroinflammation. Its aberrant activation can induce inflammatory responses involved in the pathological process of depression [39,40]. The serotonergic synapse pathway is a core pathway in depression, and its dysfunction constitutes an important neurochemical basis for the development of depression [41]. Comparison between the BEO and CCT groups showed that the differential abundant metabolites were significantly enriched in pathways including pyrimidine metabolism, taste transduction, and drug metabolism (other enzymes). In the pyrimidine metabolism pathway, pyrimidine compounds such as uridine and cytidine can act as neuromodulators involved in synaptic plasticity regulation and neuroprotection [42,43].

## 4. Discussion

In this study, a BEO beverage with satisfactory sensory quality was successfully prepared. Ostwald ripening is a common instability issue in emulsions prepared from essential oils, caused by the relatively high-water solubility of their terpene constituents [44,45]. To prevent this, BEO was co-emulsified with FSO, which consisted predominantly of long-chain triglycerides. Using single-factor tests and an orthogonal array design, the optimal formulation was determined as follows: inulin 0.5 g/50 g, milk powder 2.0 g/50 g, sucralose 0.008 g/50 g, and CMC 0.04 g/50 g. For the development of BEO products, the factor of stability should be first considered. In this study, the BEO beverage showed no visible phase separation over 4 months, indicating good macroscopic stability. While microscopic stability is assessed via particle size and zeta potential measurements, which can detect early changes such as droplet aggregation and coalescence. Particle size analysis revealed droplet aggregation at 30 days, reflecting a decline in microscopic uniformity, which may limit long-term commercial potential. To address this, future optimization could include higher-pressure homogenization, addition of natural stabilizers, or freeze-dried powder formulations. In addition, we need to think about the chemical stability of BEO during storage, which is also a limitation for long-term storage of the products.

Weight loss and suppression of spontaneous activity are typical clinical manifestations of depression. In this study, body weight changes, OFT, EPM, and MBT were used to evaluate the validity of the CCT-induced model and the intervention effect of the BEO beverage [46,47]. The results showed that, compared with the CON group, the CCT group exhibited significantly reduced body weight and pronounced depressive-like behaviors. These findings are consistent with previous reports using the CCT model to induce depressive-like behaviors in rodents [48]. Nevertheless, this model primarily captures HPA axis-related mechanisms, does not fully reflect the multifactorial nature of human depression. Therefore, while the CCT model is useful for studying certain aspects of depression, results should be extrapolated to humans with caution. After the BEO beverage intervention, body weight significantly recovered. All the above depressive-like behavioral parameters were also markedly improved, with effects comparable to those of the FLU group. Notably, compared with the CCT group, the VEH group also showed some improvement in depression-related indicators. This effect may be attributed to the presence of FSO and inulin in the beverage matrix. FSO is rich in ω-3 polyunsaturated fatty acids, which can alleviate neuroinflammation and improve depressive-like behavior [49]. Inulin may also provide mild auxiliary benefits [50]. However, the BEO beverage group showed significantly better mood-regulating effects than the VEH group, suggesting that BEO contributes additional benefits beyond the matrix components. Thus, the overall effect of the BEO beverage likely arises from synergistic interactions between BEO and the matrix.

Dysregulation of the HPA axis, monoamine neurotransmitter imbalance, and neuroinflammation are core mechanisms underlying depression [51,52,53]. In this study, CCT group showed significantly lower levels of 5-HT, DA, and NE in the prefrontal cortex compared to CON group, while BEO beverage intervention increased these neurotransmitters to near-control values. Based on these associations, it is speculated that BEO may influence serotonin receptor activity, modulate ACTH secretion, or contribute to HPA axis balance, potentially affecting the monoamine synthesis and release. However, direct evidence is lacking, and these interpretations remain speculative. Concurrently, chronic HPA axis dysregulation also induces systemic and central inflammation, which impairs hippocampal neuronal integrity and synaptic plasticity [54,55]. Elevated levels of pro-inflammatory cytokines are associated with corticosterone-induced neuronal damage and behavioral dysfunction [55]. In this study, CCT was associated with a pro-inflammatory shift, hippocampal neuronal loss, and reduced BDNF expression. BEO intervention was associated with reduced levels of pro-inflammatory cytokines, preserved hippocampal neuronal morphology, and increased BDNF expression. Given that BDNF plays a critical role in maintaining synaptic plasticity and mood regulation, it is hypothesized that BEO may exert neuroprotective effects by alleviating inflammation-driven hippocampal damage [56].

In addition to neuroendocrine and inflammatory regulation, accumulating evidence indicates that the gut microbiota and host metabolism play important roles in regulating stress-related mood disorders via the microbiota–gut–brain axis [57]. Recent studies have shown that bioactive natural products are associated with changes in gut microbiota composition and metabolic profiles in the context of depressive-like behaviors [58]. In the present study, CCT exposure was associated with reduced gut microbial diversity and altered abundance of key bacterial phyla, consistent with previously reported gut microbiota signatures associated with depressive phenotypes [59]. BEO treatment was associated with increased alpha diversity and a shift in the F/B ratio toward the CON group value. However, these changes should not be overinterpreted as definitive evidence of restoration of gut health, as increased diversity and F/B ratio shifts are not universally beneficial [38]. For example, *Desulfovibrio*, which were enriched in the BEO group, are not clearly beneficial and have been associated with inflammatory conditions in some studies [60,61]. The functional significance of these microbial changes requires further investigation. Metabolomics analysis revealed that differential metabolites in the BEO beverage group were relatively concentrated in the pyrimidine metabolism pathway. However, it is important to note that this finding is correlational and does not permit causal inference between alterations in pyrimidine metabolism and the observed behavioral improvements. Therefore, additional in-depth research is required to establish whether this pathway plays a functional role in the mechanism underlying the effects of the BEO beverage.

This study suggests that BEO beverage has the potential to alleviate depression-like behaviors. As a plant-derived essential oil formulation, BEO represents a candidate for further development as a functional food ingredient for long-term intervention. However, several limitations should be acknowledged. These include the relatively small sample size and use of only male mice, which limit generalizability; the potential confounding effect of the vehicle matrix, which has biological activity on its own; the absence of chemical stability and safety assessments. In addition, this study mainly focuses on product development and the assessment of emotional regulation effects. Therefore, detailed chemical characterization of BEO has not been performed. Future studies should include such analyses to better understand the relationship between its composition and biological activity. Despite these limitations, the formulation shows promise for mood management. Before broader application can be considered, further work is needed, including dose optimization, safety assessment, longer shelf-life testing, larger-scale sensory studies, and ultimately human clinical trials. This study provides preliminary experimental evidence for the development of a BEO-based functional beverage. It also offers a technical strategy for the application of natural plant essential oils in mood-regulating functional foods.

## 5. Conclusions

In this study, a BEO beverage was successfully prepared and optimized. In a CCT-induced depression model using only male mice, the beverage was associated with alleviation of depression-like behaviors. Exploratory observations revealed associations with changes in neuroendocrine, immune, gut microbiota, and metabolic parameters. However, this study has limitations such as a single animal model, small sample size, interference from the formula, and the lack of assessment of consumer acceptance. Moreover, the clinical efficacy and the causal mechanism of the gut–brain axis have not yet been established. Given that the VEH group itself exhibited a certain mood-regulating effect, the overall therapeutic effect of the BEO beverage should be understood as resulting from a synergistic interaction between BEO and the active components of the matrix, rather than from BEO alone. Therefore, these findings are preliminary and exploratory, and thus cannot be directly extrapolated to the clinical treatment of human depression. Nevertheless, this formulation warrants further investigation to determine whether it can be developed into a functional food for mood regulation.

## Figures and Tables

**Table 1:** Sensory Evaluation Criteria for BEO Beverages.

Parameter	Standard	Score
Appearance (20 points)	Non-uniform appearance with obvious stratification or precipitation	0–9
	Relatively uniform appearance with slight stratification or precipitation	10–14
	Uniform appearance without stratification or precipitation	15–20
Mouthfeel (30 points)	Unbalanced, with a rough texture	0–19
	Relatively balanced, with a relatively smooth texture	20–24
	Balanced, with a smooth texture	25–30
Odor (20 points)	No characteristic bergamot aroma, with off-odor	0–9
	Characteristic bergamot aroma present but relatively weak, no off-odor	10–14
	Rich characteristic bergamot aroma, natural, no off-odor	15–20
Taste (30 points)	Too sweet, unacceptable	0–19
	Relatively sweet, moderately acceptable	20–24
	Moderate sweetness, acceptable	25–30

**Table 2:** Factors and levels for the orthogonal array design.

Level	Factor			
	Inulin Addition (g/50 g)	Milk Powder Addition (g/50 g)	Sucralose Addition (g/50 g)	CMC Addition (g/50 g)
1	0.5	1	0.004	0.02
2	1.0	2	0.008	0.04
3	1.5	3	0.012	0.06

**Table 3:** Orthogonal Experiment Results.

Run No.	A: Inulin (g/50 g)	B: Milk Powder (g/50 g)	C: Sucralose (g/50 g)	D: CMC (g/50 g)	Sensory Score
1	0.5	1 g	0.004	0.02	76.60 ± 0.49
2	0.5	2 g	0.008	0.04	94.40 ± 1.62
3	0.5	3 g	0.012	0.06	81.00 ± 1.67
4	1	1 g	0.008	0.06	89.20 ± 0.98
5	1	2 g	0.012	0.02	81.40 ± 1.36
6	1	3 g	0.004	0.04	78.20 ± 1.47
7	1.5	1 g	0.012	0.04	82.80 ± 1.94
8	1.5	2 g	0.004	0.06	84.20 ± 1.17
9	1.5	3 g	0.008	0.02	82.40 ± 2.15
K1	252.00	248.60	239.00	240.40	
K2	248.80	260.00	266.00	255.40	
K3	249.40	241.60	245.20	254.40	
k1	84.00	82.87	79.67	80.13	
k2	82.93	86.67	88.67	85.13	
k3	83.13	80.53	81.73	84.80	
R	1.07	6.13	9.00	5.00	
optimum conditions	A1	B2	C2	D2	
k1	84.00	82.87	79.67	80.13	

## Data Availability

The original contributions presented in this study are included in the article. Further inquiries can be directed to the corresponding author.

## Abbreviations

The following abbreviations are used in this manuscript:

WPC	whey protein concentrate
BEO	bergamot essential oil
FLU	fluoxetine
CCT	chronic corticosterone treatment
CRH	Corticotropin-releasing hormone
ACTH	Adrenocorticotropic hormone
CORT	Corticosteroid hormone
5-HT	5-hydroxytryptamine
DA	Dopamine
NE	Norepinephrine
